# *Bacillus coagulans* T4 and *Lactobacillus paracasei* TD3 Ameliorate Skeletal Muscle Oxidative Stress and Inflammation in High-Fat Diet-Fed C57BL/6J Mice

**DOI:** 10.5812/ijpr-135249

**Published:** 2023-07-23

**Authors:** Masoumeh Shams, Fataneh Esmaeili, Samira Sadeghi, Mahsa Shanaki-Bavarsad, Shadi Sadat Seyyed Ebrahimi, Seyyed Mohammad Reza Hashemnia, Maryam Tajabadi-Ebrahimi, Solaleh Emamgholipour, Mehrnoosh Shanaki

**Affiliations:** 1 Department of Medical Laboratory Sciences, School of Allied Medical Science, Shahid Beheshti University of Medical Sciences, Tehran, Iran; 2 Department of Clinical Biochemistry, Faculty of Medicine, Tehran University of Medical Sciences Tehran, Iran; 3 Department of Neurology, Memory and Aging Center, University of California San Francisco, San Francisco, CA, USA; 4 Department of Biology, Tehran Central Branch, Islamic Azad University, Tehran, Iran; 5 Department of Clinical Biochemistry, School of Medicine, Tehran University of Medical Sciences, Tehran, Iran

**Keywords:** High-Fat Diet, Skeletal Muscle, Inflammation, Oxidative Stress, Probiotic

## Abstract

**Background:**

This study aims to investigate the effects of *Bacillus coagulans* T4 and *Lactobacillus paracasei* TD3 probiotics on skeletal muscle inflammation and oxidative stress in C57BL/6J mice fed a high-fat diet (HFD).

**Methods:**

Probiotics *B. coagulans* T4, and *L. paracasei* TD3 were administered to male C57BL/6J mice fed with HFD. The gene expression of macrophage infiltration markers, inflammatory cytokines, and oxidative stress indicators in the muscle tissue was investigated.

**Results:**

Treatment with *B. coagulans* T4 and *L. paracasei* TD3 reduced macrophage infiltration, accompanied by a decrease in the expression of monocyte chemoattractant protein-1 (MCP-1) and an increase in the expression of interleukin (IL)-10. On the other hand, *L. paracasei* TD3 decreased malondialdehyde (MDA) while *B. coagulans* T4 decreased carbonyl and increased catalase activity.

**Conclusions:**

Treatment with probiotics *B. coagulans* T4 and *L. paracasei* TD3 partially ameliorated obesity-induced skeletal muscle inflammation in HFD-fed mice.

## 1. Background

The increase in the prevalence of obesity over the past 20 years has been alarming worldwide (1). Obesity, as a complex and major metabolic disease, is caused by adipose tissue expansion due to an imbalance between energy intake and expenditure. The excess fat is stored in adipocytes, resulting in hypertrophy and hyperplasia, which has been associated with type II diabetes mellitus, fatty liver disease, insulin resistance, and chronic inflammation (2-4). In obesity, hypertrophied adipocytes overproduce various inflammatory cytokines, disrupting the cross-talk between the adipose tissue and important organs involved in metabolic processes, such as the liver and skeletal muscle (5, 6). Skeletal muscles, as one of the main metabolic tissues, have a prominent role in regulating glucose and lipid metabolism (7). Inflammatory mediators affect muscles’ sensitivity to insulin (8), among which tumor necrosis factor α (TNF-α), as well as other pro-inflammatory cytokines produced by macrophages within the adipose tissue, seems to play a key role in insulin resistance pathogenesis in obese individuals (9, 10). Obesity and a high-fat diet (HFD) can change macrophages’ phenotype from M2 (anti-inflammatory) to M1 (pro-inflammatory) (11). M1 macrophages highly express inducible nitric oxide synthase (iNOS) and pro-inflammatory cytokines (e.g., TNF-α, IL-1, IL-6), while M2 macrophages mainly express arginase1 (ARG1) and/or anti-inflammatory cytokines (e.g., IL-10) (12).

On the other hand, it has been shown that in addition to inflammation, HFD leads to oxidative stress by inducing the production of reactive oxygen species (ROS) and reducing the expression of antioxidant factors such as superoxide dismutase (SOD). The production of ROS and inflammatory factors leads to insulin resistance and diabetes (13). Recently, the role of gut microbiota has been suggested in regulating caloric intake and energy expenditure, thereby contributing to obesity and its metabolic complications (14). There is evidence that genetically obese animal models and patients with obesity have fewer *Bacteroidetes* and more *Firmicutes* in comparison with controls, boosting the energy yield from the host’s diet (14-16). Gut dysbiosis impairs the gut barrier and enhances the circulatory levels of gut-derived endotoxins like lipopolysaccharides (LPS). Lipopolysaccharides can penetrate into various organs, activating the immune system after being recognized by toll-like receptor (TLR)-4, nurturing low-grade inflammation in the gut and other organs (17, 18). Gut dysbiosis and chronic low-grade inflammation are pertinent to obesity and insulin resistance (17, 19-21).

Recent investigations have proposed that the manipulation of gut microbiota and the use of probiotics (microorganisms with beneficial biological functions) can be novel approaches to treating obesity and its related complication (20, 22). Probiotics are living microorganisms that, when administered in adequate amounts, have shown beneficial physiologic or therapeutic effects on the host (23). Probiotics have been documented to promote anti-inflammatory effects and modulate hyperphagia, weight gain, fat mass loss, and glucose tolerance (24-27).

*Bifidobacterium* and *Lactobacillus* are well-studied probiotics with beneficial effects on the health status and the ability to survive in various delivery formats during gastric transit (28, 29). *Bacillus* species have received attention in research as their spores enable them to survive in severe processing and storage conditions, as well as in the harsh environment of the gastrointestinal tract (GIT) (30).

Among *Bacillus* species, *Bacillus coagulans* was first isolated from spoiled canned milk by Hammer in 1915, attracting increasing attention since then (31). *Bacillus coagulans* is a spore-forming, gram-positive, lactic-producing, catalase-positive, and facultative anaerobic bacterium with a GRAS (generally recognized as safe) status affirmed by the US Food and Drug Administration (FDA). Animal and preclinical studies on *B. coagulans* have been focused on its beneficial effects on diverse diseases and conditions, including irritable bowel syndrome, colitis, rheumatoid arthritis, and obesity (31, 32). The results of one study showed that drinking apple vinegar in combination with *B. coagulans* improved serum lipid profile and insulin resistance and prevented hepatic steatosis induced by HFD in mice (32). *Lactobacillus* (Lactic acid bacteria (LAB)), a main component of human GIT microbial flora, is usually used as probiotics, either alone or in combination with other agents, for fermentation in the food and dairy industries. *Lactobacillus* species are non-spore-forming, gram-positive, anaerobic bacteria that have been noted to promote immunostimulatory effects, maintain the intestinal microbial balance, prevent GI infections, and nurture hypocholesterolemic effects (33-36).

Different *Lactobacillus* species have been investigated for their potential beneficial effects on obesity. For instance, *L. reuteri* has been associated with weight gain, while *L. casei* and *L. paracasei* were associated with weight loss (33). Moreover, *L. plantarum* was reported to correct plasma lipid profiles and body weight and attenuate systemic inflammation in rats with HFD-induced obesity (37).

There is evidence that the ingestion of *L. paracasei* in the long term reduces HFD-induced obesity by decreasing body weight and abdominal fat in rats (38). Another investigation on school-aged children documented that *L. paracasei* had a protective role against obesity induced by an unhealthy diet (33). In another study, data have shown that the consumption of *L. paracasei* prebiotics and synbiotics for 12 weeks reduced abdominal dysbiosis and inflammation in insulin-resistant obese male Wistar rats (39).

## 2. Objectives

Although there has been a rapid growth in studies on the effects of probiotics on metabolic abnormalities, the role of *B. coagulans* and *L. paracasei* on muscle inflammation and oxidative stress in HFD-induced obesity has not been investigated. In the present study, we hypothesized that *B. coagulans* and *L. paracasei* could attenuate skeletal muscle inflammation in mice models of HFD-induced obesity. For this purpose, we fed *B.coagulans* T4 (IBRC-N10791) and *L. paracasei* TDC3 (IBRC-M 10791) to C57BL/6J mice models of HFD-induced obesity and assessed their impacts on the levels of pro/anti-inflammatory cytokines and oxidative stress markers in the skeletal muscle tissue.

## 3. Methods

### 3.1. Animals and Diet

Animal study protocols were carried out and reported according to animal husbandry standards issued by the Care and Use of Laboratory Animals Committee of Shahid Beheshti University of Medical Sciences, Tehran, Iran (IR.SBMU.RETECH.REC.1399.235) and ARRIVE guidelines for the reporting of animal experiments. Experiments were performed on 6 - 8-week-old male C57BL/6J mice purchased from the Pasteur Institute of Iran and housed individually in cages under the temperature of 23 ± 1°C, 55% humidity, and a 12-hr light/dark cycle. After an initial acclimation period, the mice were weighed and randomly divided into either the standard chow diet (SCD) [10 kcal% fat, n = 8] or HFD [60 kcal% fat, n = 32]. The mice were fed with these regimes for ten weeks.

Afterward, mice in the HFD group were categorized into four groups (eight mice per group): HFD control group, HFD + *L. paracasei* TD3 (IBRC-M 10791) (LP, 1 × 10 ^9^ colony-forming units, CFU/ day), HFD + *B. coagulans* T4 (IBRC-N10791) (BC, 1 × 10 ^9^  CFU/ day), and HFD + *B. coagulans* T4 + *L. paracasei* TD3 (BC and LP, 5 × 10 ^8^ CFU/ day). These mice received the probiotics for another eight weeks. Lyophilized powders of *L. paracasei* TD3 and *B. coagulans* T4 were purchased from Tak Biogene Company (Iran). Water, diet consumption, and weight of animals in each group were recorded weekly.

The composition of SCD and HFD was prepared based on a research diet formulation (D12492i). The pork fat used in the study was replaced by a minced sheep tail. At the end of the experiment, all mice were sacrificed, and their tissue and blood samples were used to measure fasting blood glucose (FBG) (n = 5 per group), serum triglyceride (TG) (n = 5 per group), serum insulin level (n = 5 per group), mRNA expression level (n = 5 per group) and oxidative stress markers (n = 4-6 per group).

Blood samples were collected by a cardiac puncture; then, sera were separated and kept at -80°C for further analysis. Quadriceps muscle tissues were immediately isolated, washed with cold phosphate-buffered saline (PBS), snap-frozen in liquid nitrogen, and held at -80°C for molecular analyses. 

### 3.2. Measuring Biochemical Parameters

After blood sampling and separating sera, biochemical parameters, such as FBG and TG, were measured using enzymatic methods and kits provided by Pars Azmoon Co. (Iran). Also, insulin was measured using an ELISA kit (MBS762025, MY BioSource, CA, USA) according to the manufacturer’s procedures, and insulin resistance was determined by the homeostasis model assessment of insulin resistance (HOMA-IR) using the following formula: Fasting glucose (mmol/l) × fasting insulin (μIU/ml)/22.5 (40).

### 3.3. Measuring Oxidative Stress Markers in the Quadriceps Muscle

The thiobarbituric acid (TBA) method was used to measure malondialdehyde (MDA), a marker of lipid peroxidation, in the muscle tissue. The reaction of TBA and MDA occurs in the presence of ferric chloride and butylated hydroxytoluene, and its optical absorption is then read at 532 nm (41).

The total antioxidant capacity (TAC) of serum was evaluated based on the conversion of ferric tripyridyltriazine complex (Fe3+-TPTZ) to ferrous tripyridyltriazine (Fe2+-TPTZ) at a low pH. Then optical absorption was read at the wavelength of 593 nm. The level of protein carbonyl was measured by the 2, 4 dinitrophenylhydrazine method described earlier (42). Total thiol level in the skeletal muscle tissue was measured using 2, 2′′-dinitro- 5, 5′ dithiodibenzoic acids (DTNB) (Sigma-Aldrich) reagent, which reacts with thiol groups and forms a yellow complex with a maximum absorbance at 412 nm. We used commercial kits (SOD activity Navand Salamat, Iran) and (Catalase activity Navand Salamat, Iran) to measure manganese superoxide dismutase (MnSOD) and catalase (CAT) activity in the skeletal muscle tissue. It should be noted that the values of the above-mentioned markers were normalized based on the protein concentration of the tissues measured by the Bradford assay.

### 3.4. Quantitative PCR

Real-time quantitative PCR was performed to determine the expression of key genes responsible for macrophage infiltration into muscles [F4/80] and macrophage polarization [CD11c, CD 206, iNOS, and ARG1], as well as the genes encoding pro/anti-inflammatory cytokines [IL-1β, IL-6, IL-10, TNF-α, MCP-1], TLR2, and TLR4 in the muscle tissue. Total RNA was isolated from snap-frozen samples using a GeneAll Hybrid-R RNA purification kit (GeneAll, Seoul, Korea). First, the purity and integrity of the total RNA extracted were determined using a NanoDrop device by determining the A260/280 and A260/230 ratios. Then cDNA synthesis was performed using the RevertAid First Strand cDNA Synthesis Kit (Thermo Fisher Scientific). Quantitative reverse transcription-polymerase chain reaction (qRT-PCR) was performed using SYBR Green RealQ Plus 2 × Master Mix Green (Ampliqon) in a Real-time PCR System (Applied Biosystems, CA). The primers used have been listed in Table 1. Materials used to assess gene expression included 20 μL of PCR master mix, ten μL of SYBR Green, 1 μL of each of forward and reverse primers, 1 μL cDNA, and 7 μL of nuclease-free water. The PCR reaction was conducted in 35 cycles, and β-actin served as the internal control gene. The relative expressions of the target genes were determined by the 2^-ΔΔCT^ formula.

**Table 1.** The Sequences of the Primers Used for Real-time PCR

Target Genes	Sequence (5' - 3')	Product Length (bp)
**TNF-α**		142
F	TGCTCTGTGAAGGGAATGGG	
R	ACCCTGAGCCATAATCCCCT	
**IL-6**		138
F	GTTCTCTGGGAAATCGTGGA	
R	TCCAGTTTGGTAGCATCCATC	
**IL-1β**		76
F	TTCCTTGTGCAAGTGTCTGAAG	
R	CACTGTCAAAAGGTGGCATTT	
**IL-10**		216
F	ATGCTGCCTGCTCTTACTGACTG	
R	CCCAAGTAACCCTTAAAGTCCTGC	
**F4/80**		165
F	CTTTGGCTATGGGCTTCCAGTC	
R	GCAAGGAGGACAGAGTTTATCGTG	
**CD11c**		86
F	GCAGAGCCAGAACTTCCCAA	
R	TGCTACCCGAGCCATCAATC	
**iNOS**		199
F	TCCTACACCACACCAAAC	
R	CTCCAATCTCTGCCTATCC	
**CD206**		161
F	CCTCTGGTGAACGGAATGAT	
R	CTTCCTTTGGTCAGCTTTGG	
**ARG1**		148
F	TTGGCTTGCTTCGGAACTC	
R	GGAGGAGAAGGCGTTTGC	
**TLR2**		70
F	GCATCCGAATTGCATCACCG	
R	CCTCTGAGATTTGACGCTTTGT	
**TLR4**		119
F	TCCCTGCATAGAGGTAGTTCC	
R	TCAAGGGGTTGAAGCTCAGA	
**MCP1**		105
F	ACTGCATCTGCCCTAAGGTCTTCA	
R	AGAAGTGCTTGAGGTGGTTGTGGA	
**β-actin**		171
F	CATCCGTAAAGACCTCTATGCCAAC	
R	ATGGAGCCACCGATCCACA	

### 3.5. Statistical Analysis

Statistical analysis was performed using Prism software (GraphPad Prism 9). Data were analyzed by one-way analysis of variance (ANOVA) followed by Tukey’s multiple comparison tests at the α = 0.05 level. Data were presented as Mean ± SD, and P values < 0.05 were considered statistically significant.

## 4. Results

### 4.1. The Effects of Bacillus coagulans T4 and Lactobacillus paracasei TD3 on Obesity-induced Alterations in Biochemical Parameters

First, obesity-related characteristics were assessed in the mice receiving different diets. The HFD-fed group showed a significant increase in weight compared to the SCD group (P < 0.0001). Data revealed that *L. paracasei* and/or *B. coagulans* T4, both alone and in combination, decreased weight in the HFD group (Table 2).

Also, HFD-fed mice showed higher levels of FBG, serum insulin, and serum TG in comparison with the SCD group (P = 0.0001), indicating that *L. paracasei* TD3 and *B. coagulans* T4, either alone or in combination with each other, could reduce the levels of these metabolic parameters in mice models of obesity (Table 2).

**Table 2.** The Characteristics of Mice in Study Groups ^a^

Variables	Groups				
	SCD	HFD	HFD + *B.coagulans* T4	HFD + *L. paracasei* TD3	HFD + *B. coagulans* T4 + *L. paracasei* TD3
**FBS (mg/dL)**	110.25 ± 10.55	269.5 ± 28.43 ^b^	184.37 ± 26.84 ^c^	188.37 ± 28.16 ^c^	156.75 ± 32.21 ^c^
**Insulin (mg/dL)**	0.9240 ± 0.22	3.152 ± 0.45 ^b^	1.656 ± 0.37 ^d^	1.678 ± 0.35 ^d^	1.254 ± 0.59 ^c^
**Weight (gr)**	27.49 ± 1.26	40.96 ± 1.70 ^b^	32.15 ± 1.70 ^c^	30.57 ± 0.90 ^c^	29.72 ± 1.69 ^c^
**TG (mg/dL)**	54.5 ± 7.37	139.62 ± 14.06 ^b^	57.37 ± 12.44 ^c^	63.87 ± 4.5 ^c^	57.75 ± 7.40 ^c^

Abbreviations: SCD, standard chow diet; HFD, high-fat diet; HFD + *B. coagulans*, HFD + *Bacillus coagulans*; HFD + *L. paracasei*, HFD + *Lactobacillus paracasei*; HFD + *B. coagulans* + *L. paracasei*, HFD + *Bacillus coagulans* + *Lactobacillus paracasei*; Glu, Glucose; TG, Triglyceride.

^a^ The data are mean ± SDs (n = 5 mice per group).

^b^ P < 0.0001: Significantly different vs. SCD.

^c^ P < 0.0001: Significantly different vs. HFD.

^d^ P < 0.001: Significantly different vs. HFD.

### 4.2. The Effects of Bacillus coagulans T4 and Lactobacillus paracasei TD3 on HFD-induced Skeletal Muscle Inflammation 

**Figure 1.**
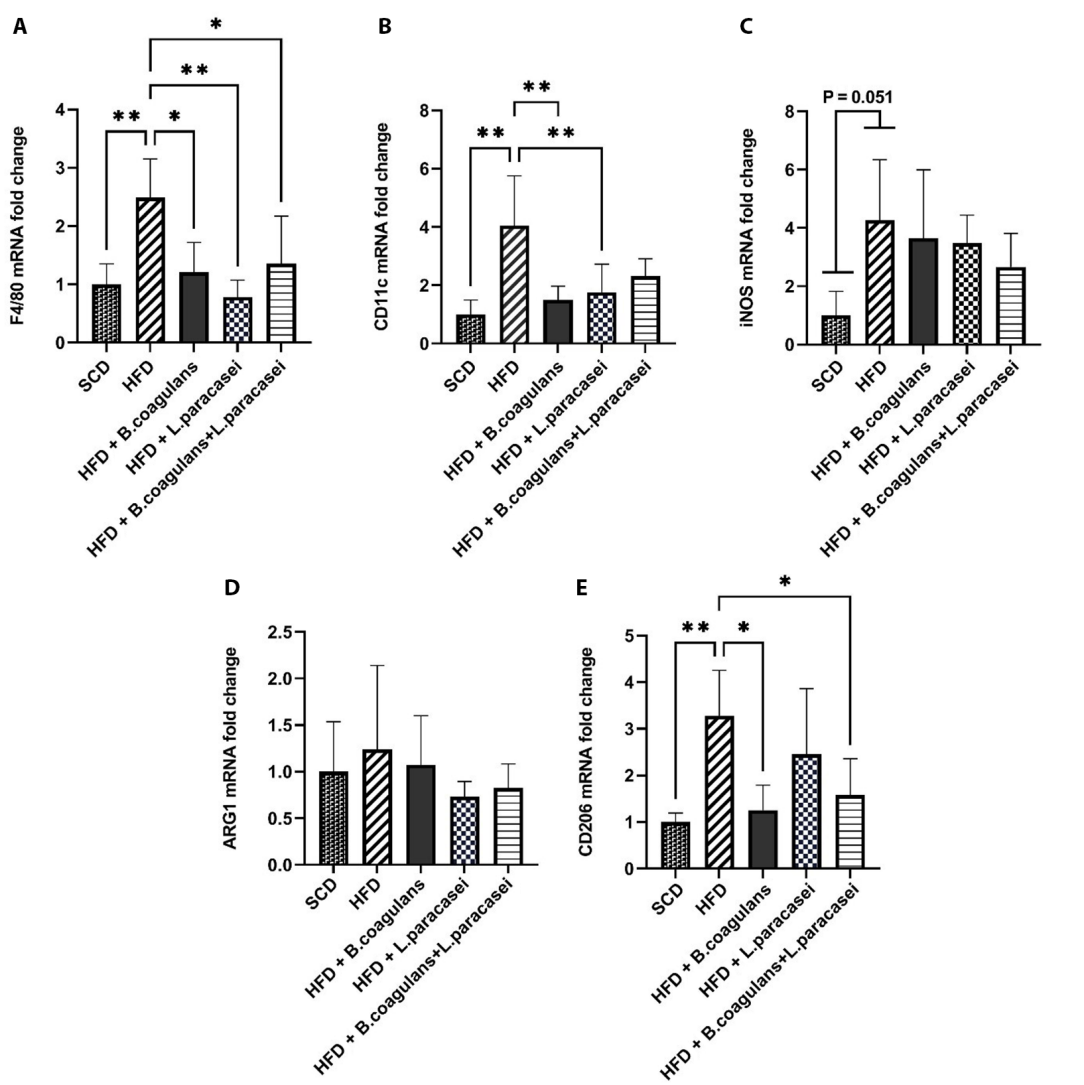
The effects of *Bacillus coagulans* and *Lactobacillus paracasei* on the marker of macrophage polarization and infiltration of the skeletal muscle: F4/80 (A), CD11c (B), iNOS (C), CD206 (D), and ARG1 (E). The bars represent mean ± SD values (5 mice per group). *P < 0.05, **P < 0.01, ***P < 0.001, and ****P < 0.0001. SCD: Standard chow diet; HFD: High-fat diet; HFD + *B. coagulans* T4: HFD + *Bacillus coagulans* T4; HFD + *L. paracasei* TD3: HFD+*Lactobacillus paracasei* TD3; HFD + *B. coagulans* T4+*L. paracasei* TD3: HFD + *Bacillus coagulans* T4 + *Lactobacillus paracasei* TD3; iNOS: Inducible nitric oxide synthetase; ARG1: Arginase1.

To determine whether *B. coagulans* T4 and *L. paracasei* TD3 affect HFD-induced skeletal muscle inflammation or not, the mRNA expressions of F4/80 (a marker for macrophage infiltration), iNOS and CD11c (a marker of M1 macrophages), as well as CD206 and ARG1 (M2 macrophages’ markers), were investigated. Our observations showed that F4/80 and CD11c expression levels in the HFD group were increased in the skeletal muscle tissue as compared with the SCD group. Furthermore, F4/80 gene expression was decreased in all three experimental groups of mice receiving probiotics (*L. paracasei* TD3, *B. coagulans* T4, and *B. coagulans* T4+ *L. paracasei* TD3) compared to the HFD control group (Figure 1A). It was noticed that *B. coagulans* T4 and *L. paracasei* TD3, alone but not in combination, reduced CD11c gene expression compared to the HFD group (Figure 1B). The polarization of macrophages from the M1 to M2 phenotype has been shown to modulate inflammation control in skeletal muscles. Our findings showed that the expression of iNOS was upregulated in the HFD group compared to the SCD group, even though the difference was marginally significant (P = 0.051, Figure 1C). Furthermore, CD206 expression level was higher in the HFD compared to the SCD group (P = 0.0037), while its expression decreased in mice receiving *B. coagulans* T4 alone and *B. coagulans* T4 + *L. paracasei* TD3 compared to the HFD control group (P < 0.05, Figure 1D). Our results also showed no noticeable change in ARG1 expression in probiotic-treated groups compared to the HFD group (Figure 1E).

**Figure 2.**
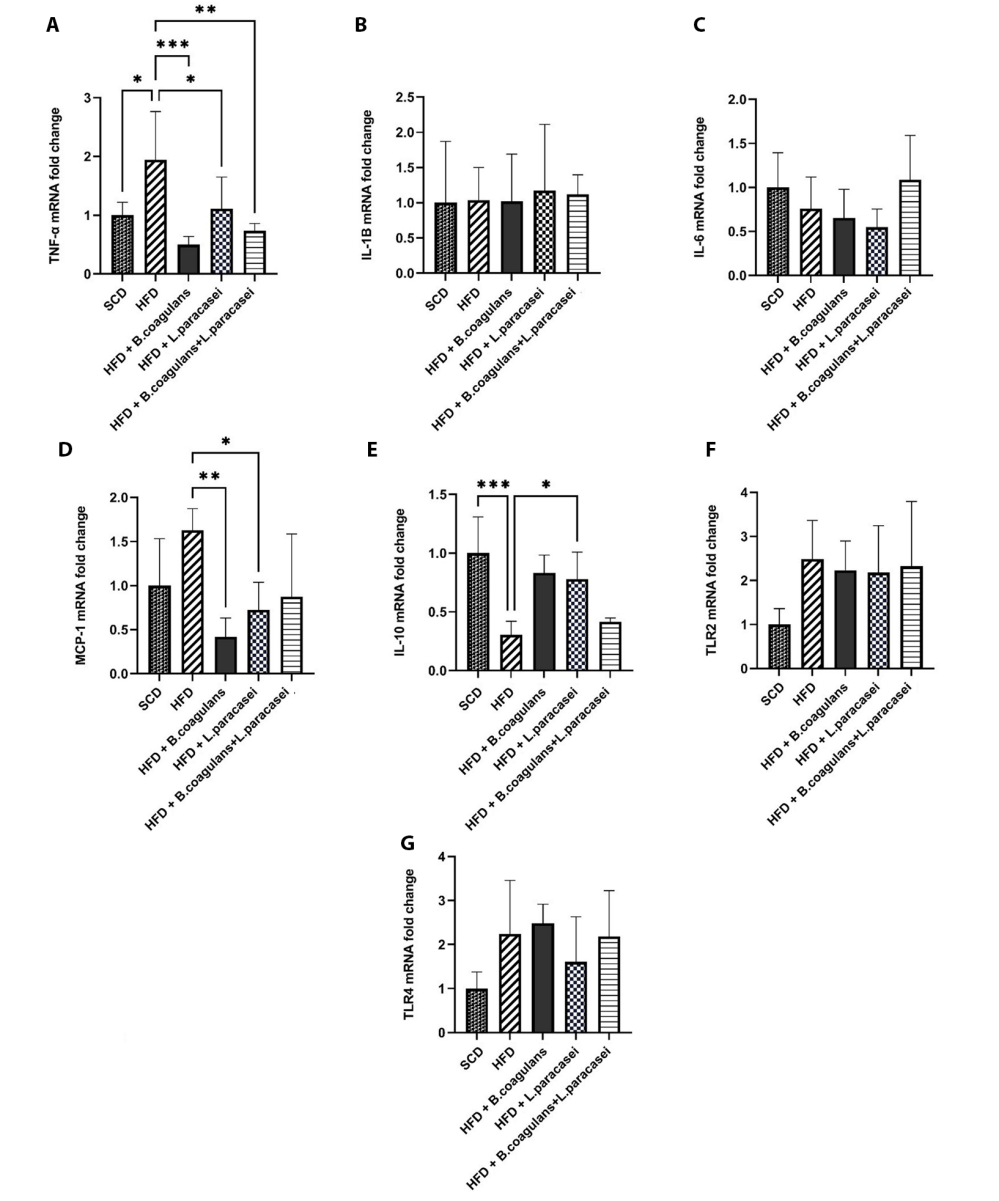
The effects of *Bacillus coagulans* and *Lactobacillus paracasei* on the gene expression of inflammatory cytokines and chemokines: TNF-α (A), IL-1β (B), IL-6 (C), MCP-1 (D), IL-10 (E), TLR-2 (F), and TLR-4 (G). Each bar represents the mean ± SD value from five mice per group. *P < 0.05, **P < 0.01, ***P < 0.001, and ****P < 0.0001. SCD: Standard chow diet; HFD: High-fat diet; HFD + *B. coagulans* T4: HFD +*Bacillus coagulans* T4; HFD + *L. paracasei* TD3: HFD + *Lactobacillus paracasei* TD3; HFD+*B. coagulans* T4+*L. paracasei* TD3: HFD + *Bacillus coagulans* T4 + *Lactobacillus paracasei* TD3; TNF-α: Tumor necrosis factor alpha; IL-1β: Interleukin-1 beta; IL-6: Interleukin-6; MCP-1: Monocyte chemoattractant protein-1; IL-10: Interleukin-10; TLR2: Toll-like receptor 2; TLR4: Toll-like receptor 4.

To determine the exact molecular mechanism behind the anti-inflammatory effects of *Bacillus coagulans* T4 and *L. paracasei* TD3, pro- and anti-inflammatory cytokines’ gene expressions were evaluated in the skeletal muscle tissue. The gene expression of TNF-α was increased in the HFD group compared to the SCD group (P < 0.05), while its expression was reduced in mice fed with either *L. paracasei* TD3 or *B. coagulans* T4 alone, as well as in the animals treated with the combination of both organisms, compared to the HFD control group (Figure 2A). There were no significant differences between the groups regarding the mRNA levels of IL-1β and IL-6 (Figure 2B and C). Also, evaluating IL-10 gene expression as an anti-inflammatory cytokine revealed its downregulation in the HFD group compared to the SCD group. In addition, IL-10 mRNA level showed a significant increase in mice treated with either *L. paracasei* TD3 or *B.coagulans* T4 alone compared to the HFD group (Figure 2E).

Our results showed that the expression of MCP-1, a crucial factor in the regulation of macrophage infiltration and recruitment, decreased significantly in the skeletal muscle tissues of the mice treated with either *B. coagulans* T4 or *L. paracasei* TD3 alone compared to the HFD group (Figure 2D). 

Moreover, neither HFD nor supplementation with the aforementioned probiotics could significantly change the transcript levels of the TLR2 and TLR4 genes in the skeletal muscle (Figure 2F and Figure 2G).

### 4.3. The Effects of Bacillus coagulans T4 and Lactobacillus paracasei TD3 on Oxidative Stress Markers in the Skeletal Muscles of HFD-fed Mice

**Figure 3.**
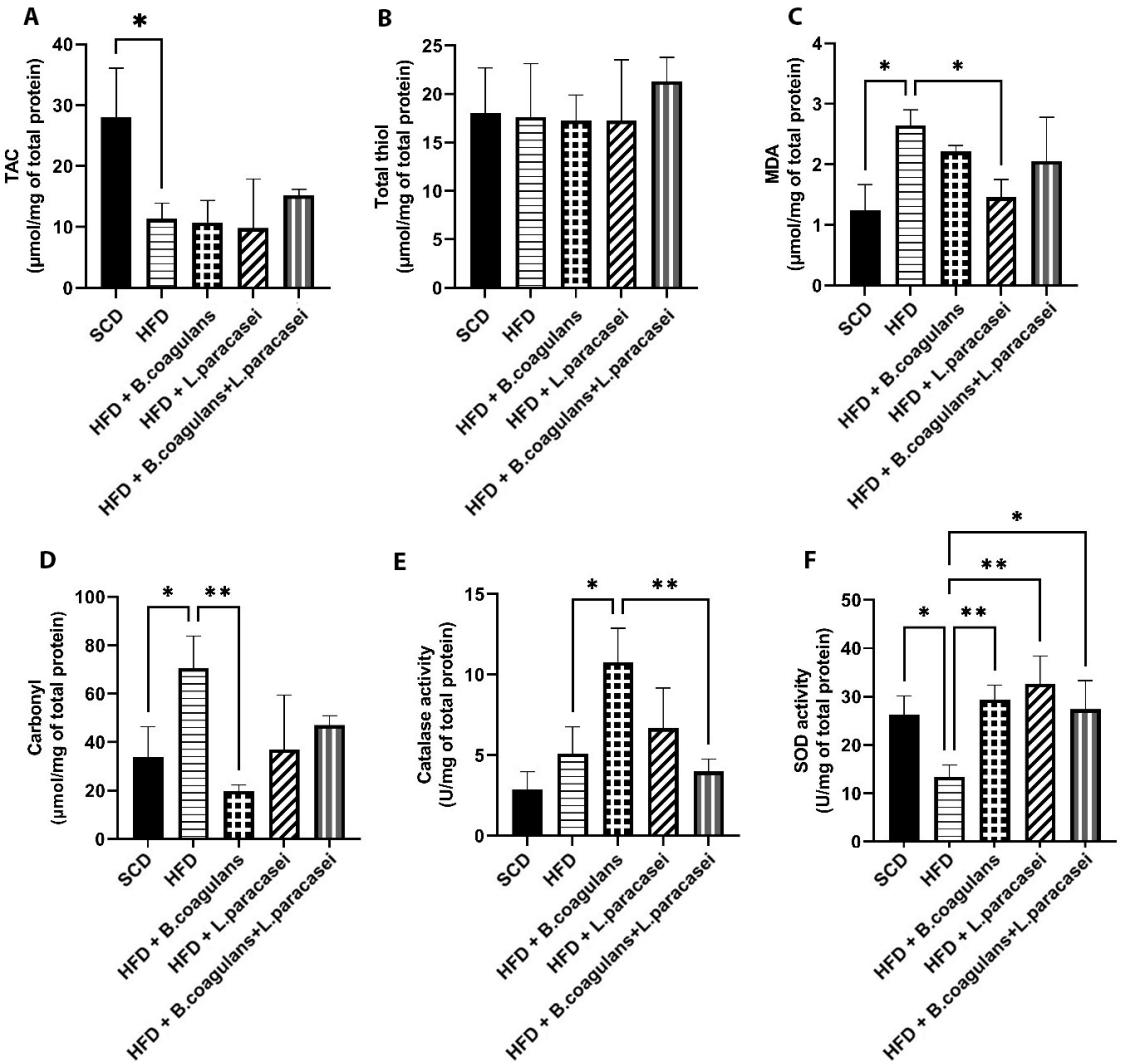
The effects of *Bacillus coagulans* and *Lactobacillus paracasei* on oxidative stress markers: TAC (A), total thiol (B), MDA (C), carbonyl (D), catalase activity (E), and SOD activity (F). Each bar represents the mean ± SD value from four to six mice per group. *P < 0.05, **P < 0.01, ***P < 0.001, and ****P < 0.0001. SCD: Standard chow diet; HFD: High-fat diet; HFD + *B. coagulans* T4: HFD + *Bacillus coagulans* T4; HFD + *L. paracasei* TD3: HFD + *Lactobacillus paracasei* TD3; HFD+*B. coagulans* T4+*L. paracasei* TD3: HFD + *Bacillus coagulans* T4+*Lactobacillus paracasei* TD3; TAC: Total antioxidant capacity; MDA: Malondialdehyde; SOD: Superoxide dismutase.

The levels of TAC, MDA, thiol, carbonyl, and the activity of SOD and catalase (CAT) were assessed in the quadriceps muscles of experimental mice to evaluate changes in oxidative stress markers. The HFD group showed significantly lower TAC levels compared to the SCD group (Figure 3A), and MDA and carbonyl levels were increased in the HFD group compared to the SCD group; however, MDA and carbonyl levels were lower in other probiotic-treated groups compared to the HFD group. Nevertheless, the difference was significant for MDA only in mice treated with *L. paracasei* TD3 and for carbonyl only in mice fed with *B. coagulans* T4 (Figure 3C and D). Catalase activity was higher in the *B. coagulans* T4-treated group than in the HFD control group (Figure 3E), and SOD activity showed a significant decline in the HFD group compared to the SCD group (P = 0.033) but a significant elevation in all three probiotic-treated groups (Figure 3F). Regarding total thiol levels, the results showed no significant differences between the study groups (Figure 3B).

## 5. Discussion

In an attempt to understand the possible ameliorative effects of *B. coagulans* T4 and *L. paracasei* TD3 on plasma oxidative stress markers and skeletal muscle inflammation, we here investigated the effects of supplementing these probiotics to C57BL/6J mice models of HFD-induced obesity. The results showed that *B. coagulans* T4 and *L. paracasei* TD3, alone and in combination with each other, significantly decreased body weight and ameliorated obesity-related oxidative and inflammatory parameters, such as serum TG, FBG, and insulin levels.

The accumulation of macrophages in the skeletal muscle leads to local and systemic inflammation following obesity, and skeletal muscle inflammation plays an important role in systemic metabolic dysfunction. In line with previous studies (43, 44), we found a significant increase in F4/80 mRNA level along with a decrease in the gene expression of CD11c in the HFD group compared to the SCD group, suggesting that HFD-induced obesity augmented macrophage infiltration, which was reversed following the supplementation of *B. coagulans* T4 and *L. paracasei* TD3, suggesting that these probiotics attenuated inflammation in the skeletal muscle tissue.

Probiotics have different effects on macrophage phenotypes. For instance, some probiotics can activate M1 macrophages, facilitating the elimination of intracellular pathogens, but others may exert an anti-inflammatory effect (45). In a study, a probiotic cocktail of *L delbruckei* sp. *bulgaricus* DWT1 and *Streptococcus thermophilus* DWT4 shifted the balance of macrophages from M2 to M1 phenotype in-vitro (46). In another investigation, *L. pentosus* var. *plantarum* C29 induces M2 macrophage in mouse hippocampal macrophages from male C57BL/6J mice (47).

Obesity and HFD augmented the expression of the TNF-α pro-inflammatory gene and reduced the expression of anti-inflammatory (IL-10) genes. In the current study, this expression pattern was not seen in other investigated genes, such as IL-1β, IL-6, MCP-1, TLR2, and TLR4, following supplementation with the HFD. Although the exact reason for this observation could not be explained in the present study, it could be speculated that treatment for 18 weeks with the HFD was not sufficient for inducing all inflammatory markers in the adipose tissue. It is noteworthy that adipose tissue inflammation is generally triggered before the development of inflammation in other tissues (48).

Regarding the impact of probiotics supplementation on inflammatory genes’ expression, our data demonstrated that *B. coagulans* T4 and *L. paracasei* TD3 alone decreased TNF-α and MCP-1 expression and increased IL-10 expression. Also, combined treatment with these probiotics only reduced the expression of TNF-α. Supporting the present data, the combined oral administration of *L. mucosae* AN1 and *L. fermentum* SNR1 decreased the plasma levels of IL-6 and TNF-α and increased IL-10 levels in the paw tissues of the rat models of acute and chronic inflammation. The discrepancy observed between our research, and the aforementioned study may be related to the fact that the expression of inflammatory genes in the recent study was measured in the local inflammation induction area (49).

In another study, the effects of *Bacillus* strains on the expression levels of TNFα, IFNγ, MCP-1, IL-12, and IL-1β were evaluated in the liver and skeletal muscle of C57BL/6J male mice, where the mice were fed with HFD for two weeks and then received probiotics for 13 weeks. The results revealed that in the liver, all cytokines decreased, but this decline was marginally significant only for IL-1β. Furthermore, the expression of MCP-1 significantly declined in the skeletal muscles of *Bacillus*-treated mice, while the decrease in the levels of TNFα, IL-1β, and IFNγ was statistically nonsignificant (50). In another study, mice were fed with HFD for three weeks, which increased the level of IL-10 protein in the skeletal muscle in the HFD group compared to the SCD group (43). It is worth mentioning that the time of initiating the treatment after HFD supplementation varies in different studies and might be the reason for inconsistencies.

It is well-established that obesity induces oxidative stress and, in turn, oxidative stress, in a vicious cycle, worsens inflammation, contributing to the development of obesity-related metabolic abnormalities (51). Here, *B. coagulans* T4, and *L. paracasei* TD3 probiotics could partly augment the antioxidant defense system and reduce oxidative stress markers, partly explaining the reduction in skeletal muscle inflammation in obese rats following oral supplementation with *B. coagulans* T4 and *L. paracasei* TD3.

It should be noted that we found no statistical difference in metabolic parameters, oxidative stress markers, and transcript levels of inflammatory genes between mice treated with the combination of *B.coagulans* T4 and *L. paracasei* TD3 and the animals receiving each probiotic alone. Hence, it can be speculated that the combined administration of *L. paracasei* TD3 and *B. coagulans* T4 had no favorable effects in comparison with when each probiotic was administered alone. There is still a great level of uncertainty about whether or not a mixture of several probiotics is more effective than a single probiotic strain. Although our results in some aspects are in line with others’ findings (49, 52, 53), discrepancies may be related to the type of probiotic strains, duration of treatment, and the dosage of the probiotics used. More studies are needed to establish the role of these parameters.

### 5.1. Conclusions

**Figure 4.**
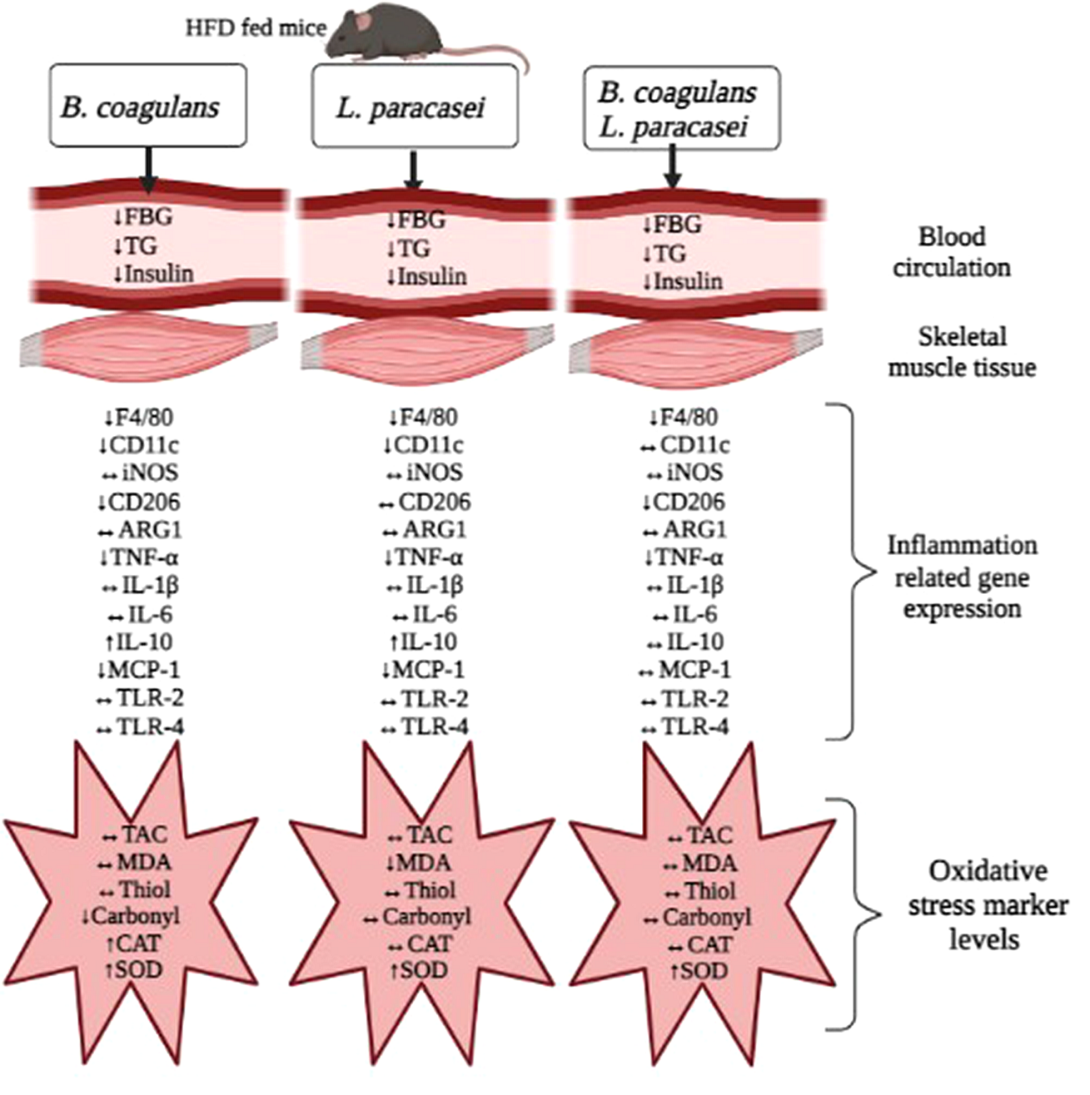
Schematic representation of the effect of *Bacillus coagulans* T4 and *Lactobacillus paracasei* TD3 on the skeletal muscle’s oxidative stress and inflammation in high-fat diet-fed C57BL/6J mice. Both alone and in combination, *B. coagulans* T4 and *L. paracasei* TD3 probiotics could partly prevent obesity-induced inflammation by suppressing macrophage infiltration, macrophage phenotype switching from M1 to M2, decreasing the production of pro-inflammatory cytokines, and enhancing the antioxidant defense system. The upward arrow indicates an increase, the downward arrow indicates a decrease, and the horizontal arrow indicates no change in comparison with the HFD-induced model. HFD: High-fat diet, FBG: Fasting blood glucose, TG: Triglyceride, TNF-α: Tumor necrosis factor-α, IL-1β: Interleukin 1β, IL-6: Interleukin 6, MCP-1: Monocyte chemoattractant protein-1, IL-10: Interleukin 10, TLR-2: Toll-like receptor-2, TLR-4: Toll-like receptor-4, ARG1: Arginase-1, iNOS: Inducible nitric oxide synthase, TAC: Total antioxidant capacity, MDA: Malondialdehyde, Mn-SOD: Manganese superoxide dismutase, CAT: Catalase.

It seems that *B. coagulans* T4 and *L. paracasei* TD3 probiotics, either alone or in combination, could partly prevent obesity-induced inflammation by suppressing macrophage infiltration, shifting macrophages’ phenotype from M1 (pro-inflammatory) to M2 (anti-inflammatory), decreasing the production of pro-inflammatory cytokines, and enhancing the antioxidant defense system (Figure 4). These results suggest that *B. coagulans* T4 and *L. paracasei* TD3 may be highly beneficial for overcoming obesity-related adverse effects.

## Data Availability

All data generated or analyzed during this study are included in this article. The raw datasets, however, are available from the corresponding author on reasonable request.
